# Veterinarian attitudes towards immunocontraceptive use in cats

**DOI:** 10.1111/avj.70113

**Published:** 2026-08-06

**Authors:** E Vartola, S Wills, E Cottingham, CD Marth, N Krekeler

**Affiliations:** ^1^ Melbourne Veterinary School, Faculty of Science The University of Melbourne Werribee Victoria Australia

**Keywords:** feline immunocontraceptives, feline population control, feline sterilisation, surgical sterilisation alternatives

## Abstract

**Background:**

The continuously expanding global cat population poses concerns to public health, native ecosystems and individual animal welfare. These concerns have led to additional management strategies to reduce unowned cat numbers, including the development of feline immunocontraceptives. Use of these contraceptives could also provide pet owners with alternatives to surgical sterilisation. However, the implementation of immunocontraceptives into feline medicine requires the support of the veterinary community.

**Aims:**

The aim of this research was to evaluate the current global attitudes and interests held by veterinarians towards immunocontraceptive use in cats to guide future research in the field.

**Methods:**

A globally distributed survey was conducted assessing veterinarians' attitudes and practices regarding surgical sterilisation and the use of immunocontraceptives in feline populations as an alternative to surgical sterilisation.

**Results:**

The survey was completed by 122 veterinarians across 11 countries. The majority, 59% of respondents, felt unfamiliar with immunocontraceptives. The main concerns included longevity of reproductive contraception (86% agreed), product efficacy (78% agreed) and cost (73% agreed). Overall, 61% of respondents reported they were more likely than unlikely to implement the use of feline immunocontraceptives should they become available in the future.

**Conclusion:**

These results indicate that the majority of responding veterinarians were interested in utilising feline immunocontraceptives should they become available. This survey highlights the need for further high‐quality research and education to improve practitioner knowledge regarding immunocontraceptive action, efficacy and safety in cats and to increase the likelihood of veterinarians utilising these therapies should they become licensed and available in the future.

## Background

Cats (*Felis catus*) have now reached all continents except for Antarctica and continue to be one of the most popular pets worldwide.^1^ Nevertheless, the proportion of cats considered stray or feral is significantly higher than those considered pets.^2^, ^3^ For the purposes of this article, community and feral cats are collectively referred to as unowned cats, that is, cats that are free‐roaming and not considered owned household pets. The success of the species can be explained by their high fecundity and adaptability in various urban and rural environments.^4^ Given the noteworthy risk to public health and vulnerable ecosystems, unowned cat populations are the target of significant global research and conservation efforts.^5^, ^6^

Increased human‐cat contact facilitates the transmission of notable zoonotic diseases, such as rabies and *Toxoplasma gondii*, especially in areas with a high density of free‐roaming cats.^6^, ^7^, ^8^, ^9^ Moreover, this species has detrimental effects on native ecosystems through direct predation and the shedding of parasites, bacteria and viruses into the environment.^2^, ^3^, ^6^, ^10^ In Australia, cats have spread across 99.8% of the landscape and remain one of the leading causes of native mammal and bird extinction.^11^ The welfare of cats in unowned populations is also of increasing public concern, despite extensive shelter and rehoming efforts in many countries.^12^

Globally, there are various population control methods currently used to combat the issue of large unowned cat populations. These involve the capturing of individual animals or the release of lethal baits into the environment, posing strong animal welfare concerns. Control methods involving the capturing of individuals can be summarized into capture, neuter and return (CNR) or capture and remove (CR) methods. CNR requires the surgical skills and facilities required to perform reproductive sterilisation prior to release back into the population. CR requires the assessment of each captured individual to evaluate the suitability for either adoption or euthanasia. The practicality and efficacy of these methods vary depending on the target population.^13^, ^14^ In the United States, CNR and CR techniques are currently estimated to cost around 14 and 16 billion dollars respectively, making these efforts unfeasible in the face of growing unowned feline populations.^15^ In addition, the contribution of abandoned pet cats to the renewal of these populations is a point of weakness in these strategies.^14^ Therefore, the need for alternative population control strategies is clearly recognized in the literature.^16^, ^17^

The use of non‐surgical sterilisation methods, such as immunocontraceptive vaccines, has been proven effective in controlling certain wild mammal populations, including elephants, white‐tailed deer, feral horses and hamsters.^18^, ^19^, ^20^, ^21^, ^22^ Given the challenges outlined above, there has been interest in the development of similar feline‐specific contraceptives.^23^, ^24^, ^25^ The ideal contraceptive agent would be safe to use and provide long‐term sterilisation with a single administration in cats. Initiatives such as the Michelson Prize & Grants (MPG) in Reproductive Biology program in the United States (2009) have sparked research into various methods including immunocontraceptive vaccines, reproductive gene modification, targeted cytotoxin delivery and gonadotropin releasing hormone (GnRH) agonist therapy.^26^ Whilst certain temporary contraceptives including megestrol acetate (MA) contraceptives and GnRH agonists are available commercially and registered for use in cats in some countries, there are no effective long‐term contraceptives currently registered by the Food and Drug Administration (FDA), the European Medicines Agency (EMA) or other global administrations.^19^, ^27^ However, there is promising emerging evidence regarding certain novel immunocontraceptives and their efficacy in providing sustained sterilisation in cats including anti‐GnRH and rabies combination vaccines, anti‐zona pellucida (ZP)/GnRH, as well as an anti‐Müllerian hormone transgene vaccine.^23^, ^24^, ^25^, ^28^, ^29^

Despite some optimistic evidence supporting the future availability of feline immunocontraceptives and their potential role in combatting the global challenge of unowned cats, the opinion of veterinary practitioners on the matter remains unclear. As with any new medical measure, there are concerns associated with the potential introduction of feline immunocontraceptives. These concerns involve the duration of action, side effects, reversibility, administration site reactions, overall cost‐effectiveness, owner approval and behavioral changes associated with immunocontraceptive vaccines.^30^, ^31^, ^32^ The successful use of feline immunocontraceptives in both unowned population management and pet care settings requires the support and confidence of veterinary clinicians. As first‐line professionals, it is therefore important to understand veterinarians' concerns and attitudes in order to evaluate the future direction of the development of feline immunocontraceptives and their potential use in clinics. Therefore, the aim of this study was to evaluate veterinarians' attitudes and beliefs toward immunocontraceptive use in cats and the decision‐making process behind current surgical sterilisation methods.

## Methods

A cross‐sectional survey was conducted using the online platform Qualtrics (Qualtrics, Inc., Provo, UT, USA). The survey was distributed to veterinarians globally between the 22 August 2022 and 22 September 2023. The exact number of invited participants could not be determined but is estimated to be around 900. Participation in the survey was voluntary and anonymous. Written consent was obtained from each participant prior to beginning the questionnaire online. Inclusion criteria included being a registered veterinarian and being able to read and write in English. The research proposal was approved by the University of Melbourne's Human Research Ethics Committee (approval ID: 23639).

### Distribution and participant recruitment

The survey was distributed to participants via listserv emails to national veterinary associations worldwide and handing out QR‐code flyers at veterinary conferences. Survey invitations included a brief description of the study and a Plain Language Statement in English.

### Questionnaire

The survey questionnaire included a brief description of immunocontraceptives and their potential role in feline population management, followed by 19 multiple choice questions (see Appendix S1). These questions were aimed at collecting demographic information and assessing participants' current surgical sterilisation practices and familiarity with immunocontraceptives. Attitudes towards feline immunocontraceptives were further assessed through a series of questions regarding the perceived benefits and concerns associated with their use. Participants answered these questions by ranking statements on a seven‐point Likert scale, with 1 = strongly disagree and 7 = strongly agree. Some questions involved ranking statements on a 10‐point Likert scale, with 1 = extremely unlikely and 10 = extremely likely.

### Data analysis

Data collected from the survey was stored in an Excel spreadsheet on a password protected server. Upon completion of the survey, data was exported from Qualtrics into Microsoft Excel (2016) for cleaning and initial processing. A total of 154 respondents opened the survey, while 122 responses were deemed suitable for analysis. The remaining responses were excluded due to participants withdrawing at the consent stage or before answering the questions related to their attitudes towards immunocontraceptive use in cats. Descriptive analysis of respondents' demographics, awareness, knowledge and attitudes was conducted using R statistical software.^33^ Graphs were created with the R packages Likert,^34^ ggplot2^35^ and patchwork.^36^

## Results

There were 122 veterinarians that completed the majority of the survey. Some participants did not answer every survey question; therefore, the total responses are reported for each question.

Approximately 85% of survey respondents were from Australasia, with the remainder from Europe, North America and Africa (Figure 1). Most participating veterinarians graduated veterinary school between 1980 and 1989 (n = 33), with 10 participants graduating between 1970 and 1979. Twenty‐eight participants graduated during the last decade (2010–2019), 22 between 2000 and 2009 and 19 between 1990 and 1999. Nine participants graduated since 2020.

**Figure 1:**
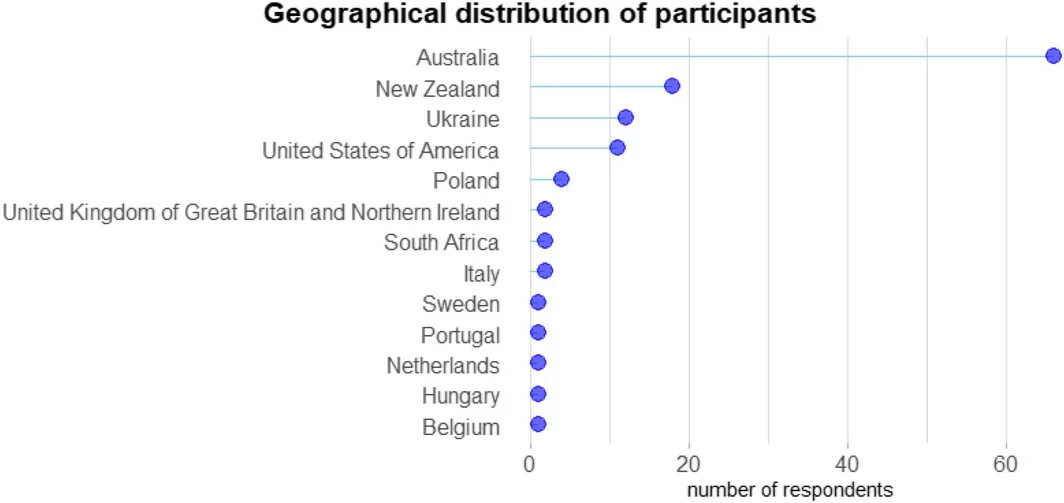
The geographical distribution of participating veterinarians based on their practicing country, 122 total survey respondents.

### Current surgical sterilisation practices

While 115 participants only chose orchiectomy as their preferred method of surgical sterilisation for male cats, six additional veterinarians reported using both orchiectomy and contraceptive implants or vasectomy. Regarding the preferred method for surgical sterilisation for female cats, 107 participants reported using ovariohysterectomy and 13 ovariectomy, either exclusively or in combination with both techniques +/− other techniques, such as hormonal contraceptives (n = 4).

Most participants agreed or strongly agreed (rankings 5–7) that the reason they chose orchiectomy for surgical sterilisation of male cats and ovariohysterectomy or ovariectomy for surgical sterilisation of female cats was because this method was taught to them at vet school and because this was the standard practice at the participant's clinic (see Figure 2 for details).

**Figure 2:**
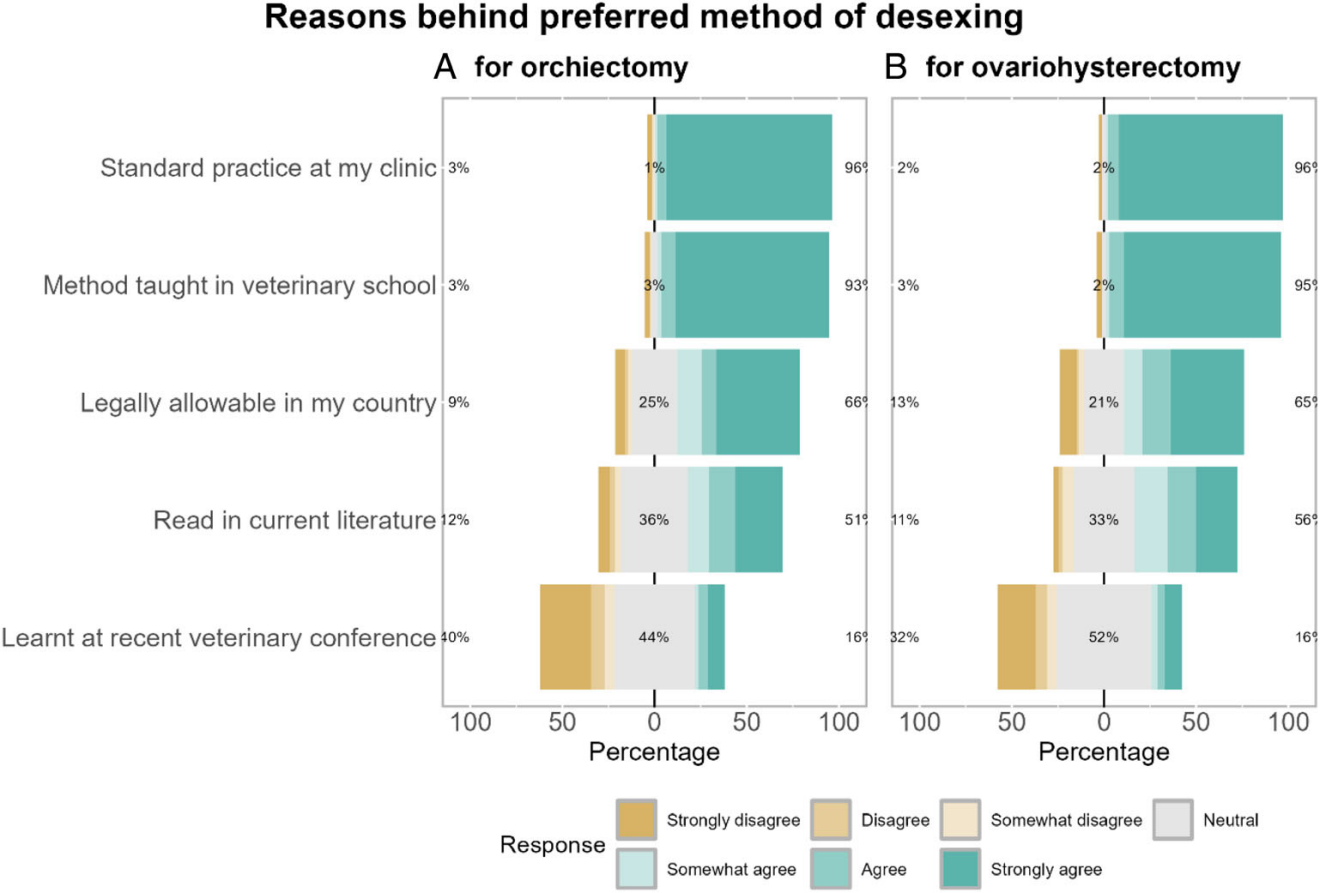
Reasons behind choosing (A) orchiectomy or (B) ovariohysterectomy as the preferred method of surgical sterilisation of cats. Participants ranked statements on a seven‐point Likert scale. The results are presented as percentages (n = 115, for A) and (n = 101, for B). The percentage labels in the graph represent summarised disagreeing responses (strongly disagree to somewhat disagree) on the left, unsure in the middle and agreeing responses (somewhat agree to strongly agree) on the right.

When asked to evaluate some of the reasons why the veterinarians thought owners may elect not to surgically sterilise their pets, personal choice, the desire to breed and cost of procedure were ranked the highest by the participating veterinarians (see Figure 3 for details).

**Figure 3:**
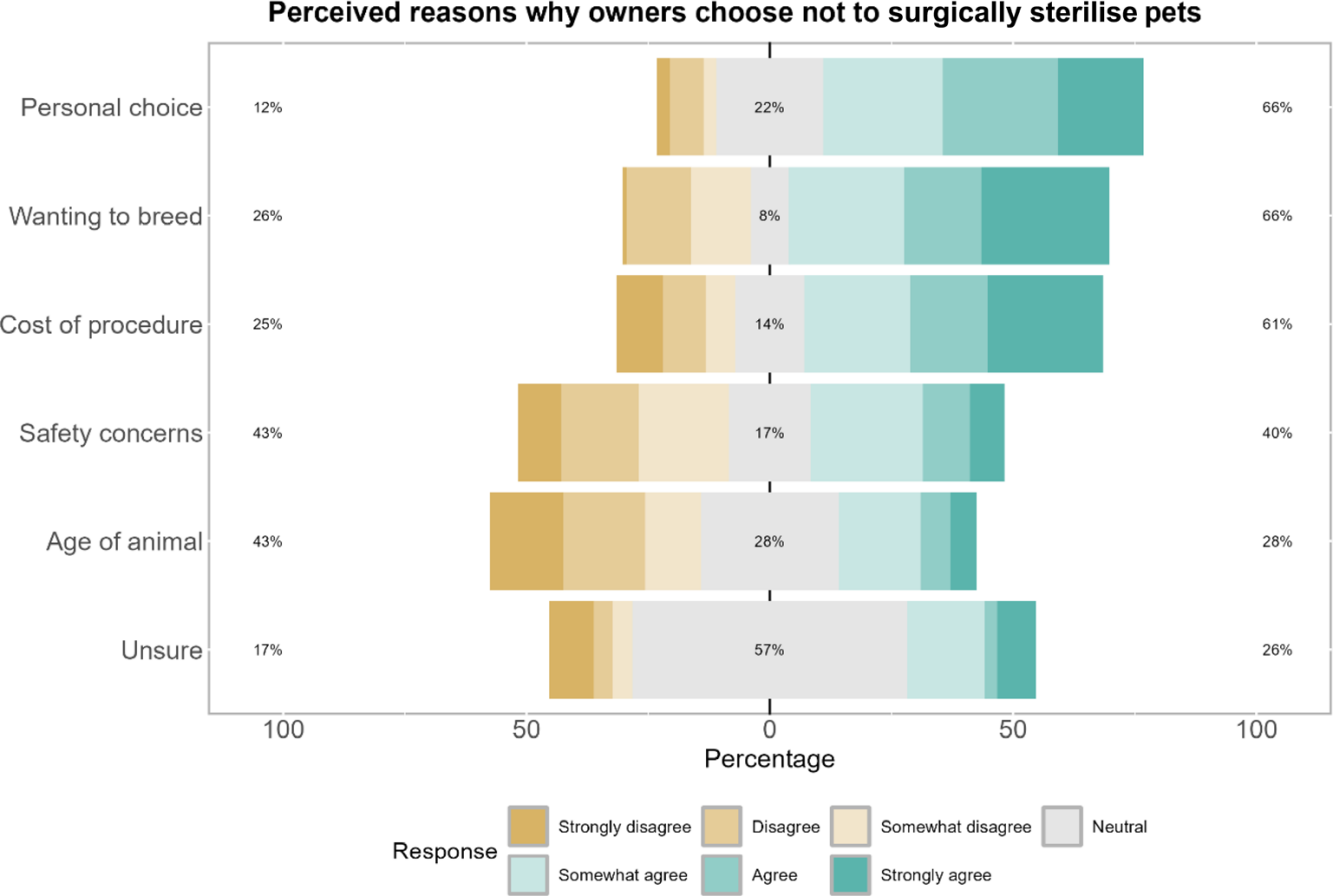
Reasons owners elect not to surgically sterilise their pets as perceived by veterinarians. Statements were ranked on a seven‐point Likert scale. The results are presented as a percentage of the total responses for each item (n = 113 or 114 for all categories except ‘unsure’ where n = 76). The category ‘other’ was excluded from the graph due to low response rates (n = 15). The percentage labels in the graph represent summarised disagreeing responses (strongly disagree to somewhat disagree) on the left, unsure in the middle and agreeing responses (somewhat agree to strongly agree) on the right.

### Attitudes towards feline immunocontraceptives

Most respondents felt unfamiliar with immunocontraceptives in general (1–4/10: 59% [72/122]). The remaining 32% (n = 39) considered themselves familiar (6–10/10) and 9% (n = 11) neither familiar nor unfamiliar (option 5/10). When asked to consider in which context they could see immunocontraceptives playing a beneficial role, 86% of participants selected ‘performing surgical sterilisation without general anesthesia, followed by 83% selecting “use in stray cat populations to avoid need for surgical desexing resources” (see Figure 4 for details).

**Figure 4:**
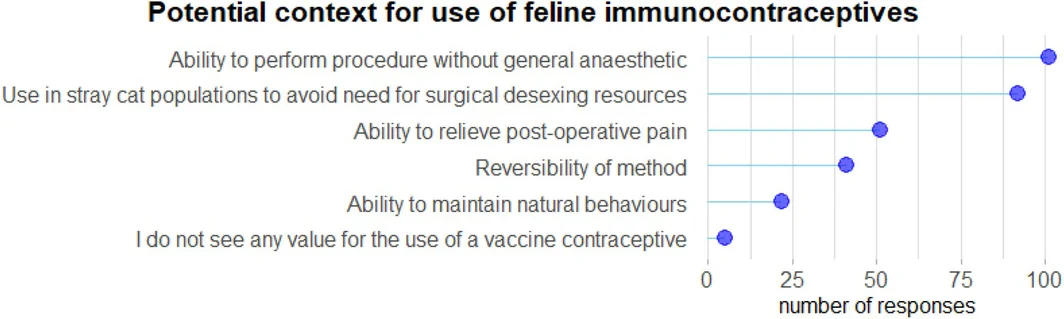
The context in which participating veterinarians agreed feline immunocontraceptives could play a beneficial role. The results are represented as the number of times each statement was selected with a total of 318 responses, noting that multiple statements could be selected by the same respondent. The option ‘other’ was selected 6 times but is not represented in the graph.

### Concerns regarding novel immunocontraceptives in feline use

Participants had the strongest concerns about longevity of the contraceptive effect and product efficacy when asked to consider reasons for opting not to use immunocontraceptives, should they become available and licensed for use in cats (see Figure 5 for details). Additional concerns (n = 14) listed as free text included side effects affecting feline health or behavior and the safety of viral vectors.

**Figure 5:**
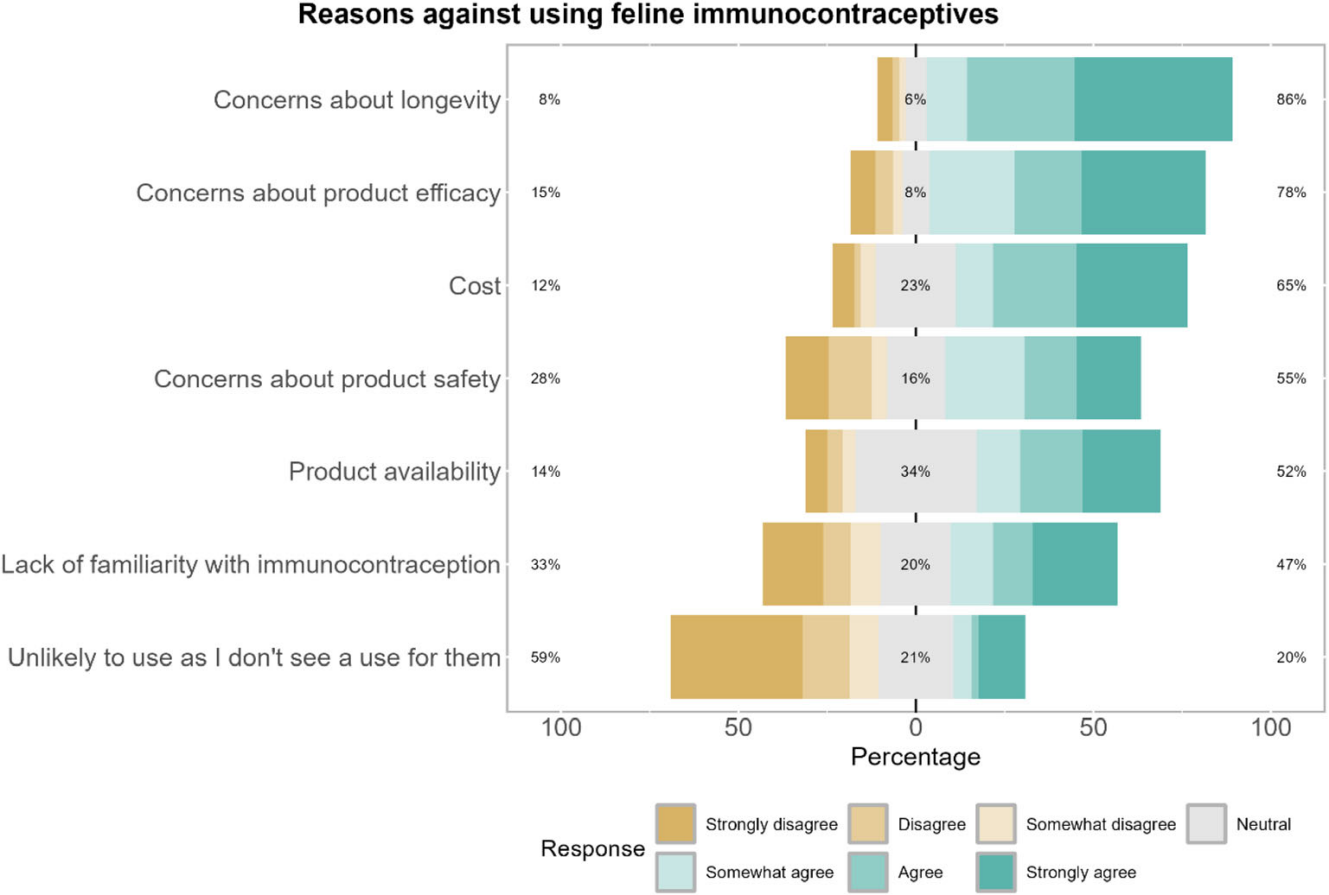
The main reasons participating veterinarians would choose not to utilize immunocontraceptives over traditional surgical methods should they become licensed and available to use in cats. Participants ranked statements on a seven‐point Likert scale. The results are presented as a percentage of the total responses for each item (n = 114–117 for all responses, except ‘unlikely to use as I don't see a use for them’, where n = 99). The category ‘other’ was excluded from the graph due to low response rates (n = 14). The percentage labels in the graph represent summarized disagreeing responses (strongly disagree to somewhat disagree) on the left, unsure in the middle and agreeing responses (somewhat agree to strongly agree) on the right.

The most important features that would make veterinarians choose immunocontraceptives over surgical or other sterilisation methods included easier implementation, reduced morbidity and improved efficacy (see Figure 6 and Appendix S2 for details). Cost effectiveness to the client was considered more important than cost effectiveness to the veterinary clinic. The respondents considered suppressing sexual behaviors as an important part of routine surgical sterilisation, with most considering it more important in male than female cats (Figure 7).

**Figure 6:**
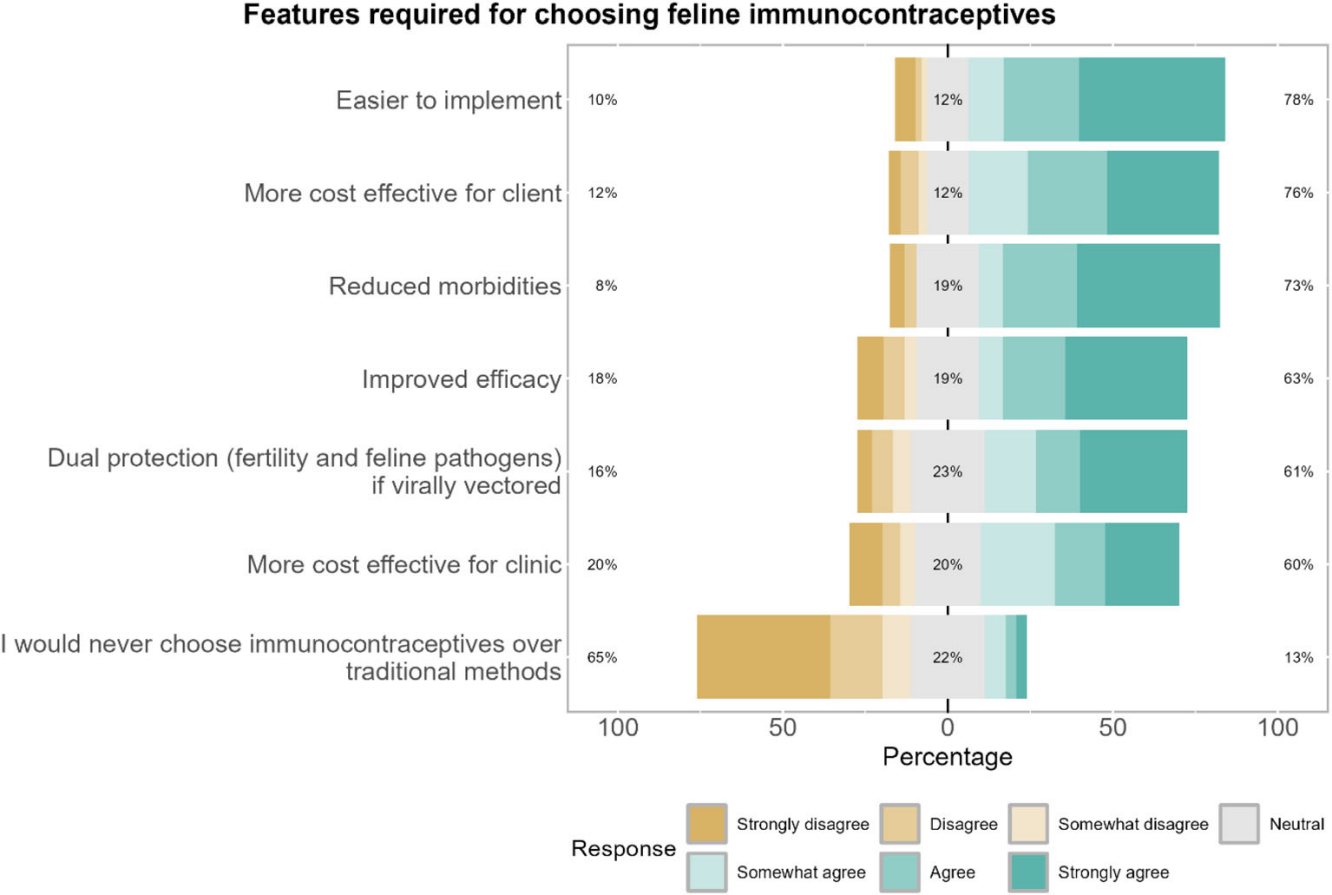
Major features of feline immunocontraceptives required to make participating veterinarians choose to use them over traditional surgical sterilisation methods. Participants ranked statements on a seven‐point Likert scale. The results are presented as a percentage of the total responses for each item (n = 111–113 for all responses, except ‘I would never choose immunocontraceptives over traditional methods’, where n = 94). The category ‘other’ was excluded from the graph due to low response rates (n = 12). The percentage labels in the graph represent summarised disagreeing responses (strongly disagree to somewhat disagree) on the left, unsure in the middle and agreeing responses (somewhat agree to strongly agree) on the right.

**Figure 7:**
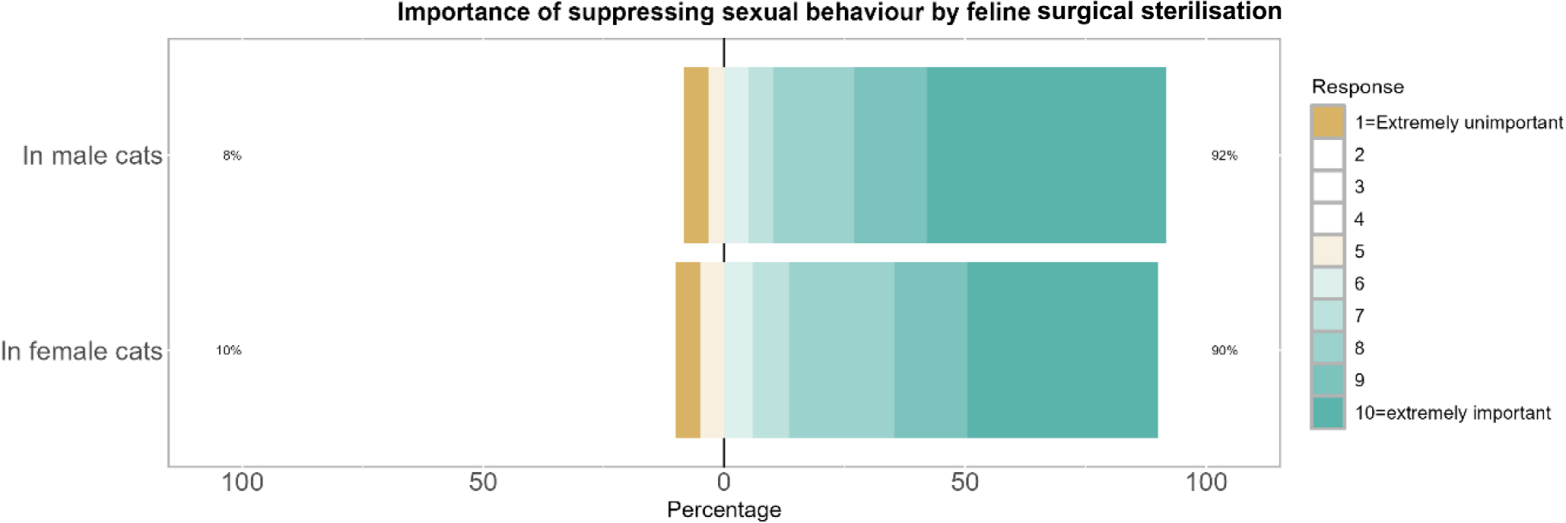
Participants' perceived importance of suppressing or avoiding sexual behaviors in routine surgical sterilisation of male and female cats, on a scale of 1 being extremely unimportant to 10 being extremely important. The results are presented as a percentage of a total of 119 responses for each item.

In regard to major concerns, most respondents (61%) felt strongly about the possibility of waning efficacy. Other concerns included as free text responses involved the development of other immune‐mediated diseases as a consequence of immunocontraceptive administration (n = 7). Still, participating veterinarians responded that they were more likely than unlikely to use an immunocontraceptive should one become licensed and available for use in cats (Figure 8A). Drivers that were reported to help with future uptake were increased amounts of published peer‐reviewed literature on feline immunocontraceptives and the ease of their use (see Figure 8B for details). Participants who included the statement ‘other’ in their answer provided written answers regarding cost‐effectiveness and proven efficacy with single administration. When asked to evaluate the likelihood of owner and breeder acceptability of immunocontraceptives, somewhat more participants considered it likely (48% and 46%, respectively) or were neutral on the matter (34% and 26%, respectively), than unlikely (18% and 26%, respectively).

**Figure 8:**
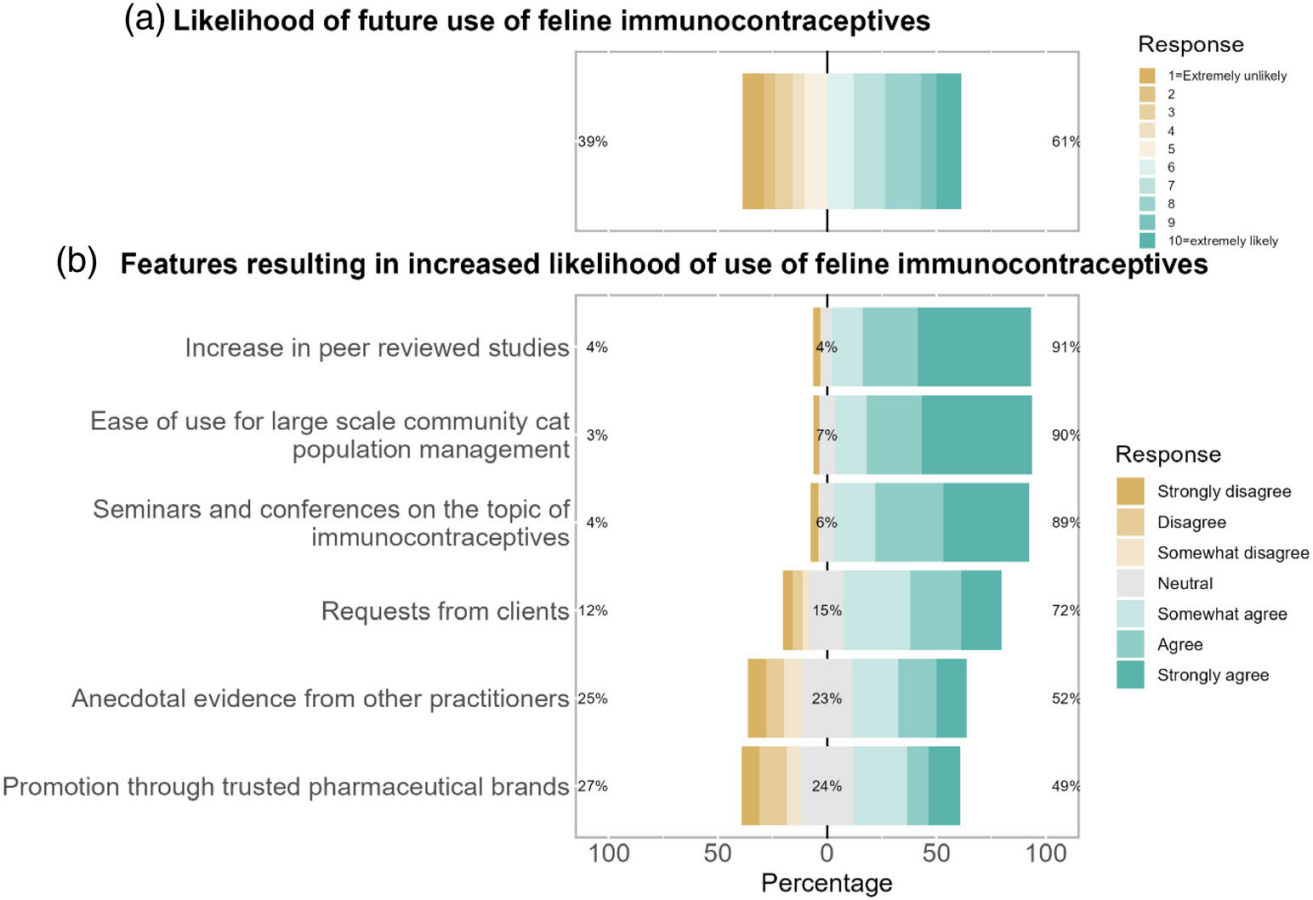
Participating veterinarians ranked (A) the overall likelihood of using feline immunocontraceptives in the future (on a 10‐point scale from extremely unlikely to extremely likely) and (B) features which would increase their likelihood of using feline immunocontraceptives in the future should they become available and licensed (on a seven‐point Likert scale). All results are presented as a percentage of the total responses for each item (n = 109–114 for all responses). The category ‘other’ was excluded from the graph due to low response rates (n = 10). The percentage labels in the graph represent summarised disagreeing responses (strongly disagree to somewhat disagree) on the left, unsure in the middle and agreeing responses (somewhat agree to strongly agree) on the right.

## Discussion

This survey was conducted to assess veterinarian attitudes towards immunocontraceptives as a feline reproductive control measure. The aim of the study was to collect information on the decision‐making behind current surgical sterilisation practices and to gauge veterinarians' knowledge and confidence regarding immunocontraceptives in light of their potential application in the future. The results of this study showed that there was considerable interest in feline immunocontraceptives among the responding veterinarians, despite unfamiliarity with these novel contraceptives and persisting concerns regarding their efficacy and practicality. The low estimated overall response rate of less than 15% of invited veterinarians raises some concerns about the representativeness of the sample. This limited response may have introduced biases, as those veterinarians with stronger opinions or direct experiences with feline immunocontraceptives might have been more likely to respond, while those less experienced or interested may have opted not to engage. Further engagement with the veterinary community will become necessary should immunocontraceptives become more widely available to ensure veterinarians are comfortable and competent in using these products.

Current research regarding feline immunocontraceptives has centered around investigating the reproductive effects of various novel immunocontraceptive methods in female or male cats.^23^, ^28^, ^29^, ^31^, ^32^, ^37^ A study by Levy *et al*. reported 93% of 15 queens vaccinated with an anti‐GnRH vaccine remaining infertile for the first year following treatment.^23^ Another study by Vansandt *et al*. reported successful suppression of ovulation and pregnancy rates with a single administration of a novel anti‐Müllerian hormone transgene vaccine in six female cats for at least 2 years, with no adverse local or systemic reactions.^37^ Despite promising evidence on the matter, the need for longer‐term and larger‐scale feline population studies was evident in the current literature.

To the best of our current understanding, the present study is the first of its kind to assess veterinarians' attitudes towards immunocontraceptive use in cats and may prove valuable in the future to assess shifting attitudes in this emerging field. Notably, the majority of participating veterinarians felt unfamiliar with immunocontraceptives in general. This may be expected due to their relative novelty in small animal medicine, despite their registered use in other mammalian species^18^, ^19^, ^20^, ^21^ and may also reflect differences in clinicians' years of experience or time since graduation. Despite the unfamiliarity, a majority of participating veterinarians in this study (61%) were interested in utilising immunocontraceptives should they become available and licensed for use in cats in the future. The adaptation of immunocontraceptives as a sterilisation strategy could significantly aid unowned cat population management by providing cost‐effective and non‐invasive alternatives to current population controlling techniques.^14^, ^30^, ^38^ In addition, their commercial availability would provide pet owners with alternatives to surgical sterilisation and the associated cost. As evident by the results presented in Figures 3, 5 and 6, cost remains an important factor in veterinary and perceived client decision‐making.

The current preferred surgical sterilisation methods reported in this study were in line with expected results. Surgical sterilisation remains the global standard for reproductive sterilisation of cats. The reasons behind these choices are strongly influenced by veterinary clinic protocols and primary veterinary education (Figure 2). It could be speculated that the success of future feline immunocontraceptives in both population‐control and commercial markets requires the acceptance, if not promotion, of these modalities by veterinary practitioners and in veterinary education settings. Notably, the majority of veterinarians participating in this study felt that increased amounts of peer reviewed literature and information gained at veterinary conferences or seminars would increase their likelihood of choosing feline immunocontraceptives in the future. This highlights the demand for additional high‐quality evidence from larger‐scale studies in order to address veterinarians' concerns regarding the practicality, long‐term efficacy and safety of the products, as shown in Figure 6. Specifically, increased evidence to demonstrate reduced morbidities in comparison to surgical sterilisation methods, improved cost‐effectiveness and addressing possible adverse consequences associated with immune‐modulatory therapies were deemed as important factors requiring a larger evidence base. As shown by the results of this survey, improved access to this information and additional education at veterinary events are required to improve veterinarians' familiarity and confidence regarding immunocontraceptive use. In addition, the role veterinary schools can play in promoting these novel techniques is highlighted by the fact that the majority of participating veterinarians currently employ the sterilisation technique taught to them during veterinary school.

The majority of responding veterinarians saw feline immunocontraceptives playing a beneficial role in providing reproductive sterilisation without the risk of general anesthesia and a tool to be used in stray cat populations to avoid the need for surgical resources (Figure 4). Long‐term efficacy and practicality in wild populations still remain a concern for many respondents, as highlighted by Figure 5 and current literature.^23^, ^30^, ^31^, ^32^, ^37^ Providing pet owners with cost‐effective and non‐surgical alternatives could indirectly benefit population control of unowned cats by reducing the proportion of fertile pet cats that may become abandoned or are allowed to free‐roam, therefore contributing to the growth of unowned cat populations. This is especially important in countries, such as Australia, where unowned cat populations pose a significant management challenge.^5^, ^11^ Despite concerns regarding the longevity of feline immunocontraceptives as reported in Figure 5, 34% of survey respondents could imagine feline immunocontraceptives to be beneficial in the context of reversible sterilisation. Further research is necessary to clarify whether veterinarians' concerns regarding longevity are related to the potential long‐lived contraceptive effects being viewed as an advantage or disadvantage. More specifically, the potential irreversibility of immunocontraceptives' effects may be viewed as a benefit in some settings, such as in controlling unowned cat populations in remote settings, but a disadvantage in others, where veterinarians or owners may prefer alternatives that offer temporary effects on fertility in comparison to irreversible surgical sterilisation.

It is important to note that the participants in this study may not represent veterinarians as a global collective. The geographical distribution of participants was dominated by Australia and New Zealand (Figure 1) and limited to veterinarians practicing in a total of 11 countries despite efforts to distribute the survey globally. The opinions and beliefs regarding feline immunocontraceptives and current surgical sterilisation practices represented by the results of this study may vary greatly in other geographical locations or sectors of the profession that were not reached by the current survey. Indeed, veterinarians in locations where immunocontraceptive use is more prevalent to control wildlife populations, such as the United States^19^ or South Africa,^33^ may be more familiar with the technology and consequently provide alternative insights that could improve immunocontraceptive research and uptake. Making this survey available to more diverse veterinary communities, particularly those with large unowned cat populations that are impacted by the zoonotic and environmental risks associated with them could significantly aid in improving the design of future feline immunocontraceptives. Nevertheless, the results of this survey provide insights into the current concerns and beliefs regarding immunocontraceptives in the veterinary community as well as promising pathways to dissemination of new knowledge.

## Conclusion

In conclusion, this study shows that veterinarians would be interested in utilising feline immunocontraceptives as reproductive control methods in cats, should they become available and licensed for felines in the future. However, further peer‐reviewed research on the long‐term safety and efficacy, cost‐effectiveness and ease of use of potential feline immunocontraceptives are required before the veterinary community would be willing to adopt the technique routinely.

## Conflicts of interest and sources of funding

The authors declare no conflicts of interest or sources of funding for the work presented here.

## Supporting information


**Appendix S1.** Assessing veterinarian attitudes towards immunocontraceptive use in cats.

**Appendix S2. Figure 3.** Reasons owners elect not to surgically sterilise their pets as perceived by veterinarians.
**Figure 4.** The context in which participating veterinarians agreed feline immunocontraceptives could play a beneficial role.
**Figure 5.** The main reasons participating veterinarians would choose not to utilize immunocontraceptives over traditional surgical methods should they become licensed and available to use in cats.
**Figure 6.** Major features of feline immunocontraceptives required to make participating veterinarians choose to use them over traditional surgical sterilisation methods.
**Figure 8b.** Features which would increase their likelihood of using feline immunocontraceptives in the future should they become available and licensed.

## Data Availability

The data that support the findings of this study are available on request from the corresponding author. The data are not publicly available due to privacy or ethical restrictions.
