# Supplementary material for: Veterinarian attitudes towards immunocontraceptive use in cats
Source: Aust Vet J. 2026 Aug 6;104(10):701–9. doi: 10.1111/avj.70113 (PMC13631546; doi:10.1111/avj.70113)
Supplement: Supplementary file 1 [file AVJ-104-701-s001.docx]

# ‘Other responses’

### Fig 3. Reasons owners elect not to desex their pets as perceived by veterinarians.

- Believe it unhealthy for the cat
- “Not natural”
- Not taking ownership eg semi-owned cats, strays etc
- just not getting around to desexing.
- Anthropomorphism
- internet says don’t do it!
- not wanting the animal’s personality to change
- Cultural reasons
- Unable to catch / handle the cat
- disorganised/negligence
- unnatural style of life
- Apathy
- long term health concerns which is not the same as safety concerns which refers to animal dying when performing procedure or gets complication due to surgery
- few people in private practice don’t want their cats desexed. If so usually breeders or have a personal ethic. Q14 below really depends on the product safety, reliability and cost. Q15 will really depend on reversibility and perceptions of risk if it can’t be guaranteed.
- Anaesthetic risk

### Fig 4. The context in which participating veterinarians agreed feline immunocontraceptives could play a beneficial role.

- In breeding females to select when to breed
- to be utilised in catch and release programs in particular environments
- eradication of feral cat populations
- limited use in clinical practice
- concept of least intervention to achieve sterility
- some of these questions are benefits eg. reversibility, how this may also be a concern when comparing it to surgical methods for guaranteed reproduction control. Natural behaviours being maintained is desirable from a welfare and natural living situation. I work at an Animal Welfare practice associated with a shelter and we struggle to find foster carers for entire cats that if maintain natural behaviours due to spraying and fighting. If these behaviours were to persist post vaccination in a % of animals commitment to this method of control maybe undesirable for some owners of pet cats.
- Potential to trap and treat much larger numbers more quickly.

### Fig 5. The main reasons participating veterinarians would choose not to utilize immunocontraceptives over traditional surgical methods should they become licensed and available to use in cats

- Ability to be transmitted would need to be considered - eg viral vector
- concerns about effect on behaviour or health issues
- frequency that i have to repeat the product so intervals of administering the immuno contraceptive and also variability in duration of efficacy in different individuals. owners are going to expect 100% efficacy whilst in populations where we are currently using immunocontraceptives e.g. elephants we do not achieve 100 % efficacy despite recommended re-administration intervals but in feral populations a 90% efficacy and prevention of pregnancy is acceptable whereas for client owned cats that would not be acceptable.
- Have not reviewed the literature answering questions in order of priority.
- Possible need for confirmation of efficacy following administration (i.e. follow-up hormone level testing) - would be difficult to enforce compliance
- Possible side effects eg immune-mediated diseases
- if supported by a virus how be sure to not disseminated it, feral cats are useful against rats and mice

### Fig 6. Major features of feline immunocontraceptives required to make participating veterinarians choose to use them over traditional desexing methods.

- I cannot answer due to lack of knowledge in this field regarding efficacy, safety and frequency of vaccine implementation. I do believe however similar with human repro/medicine, surgical is the only 100% effective means for preventing unwanted pregnancies
- separate to pathogen vectored means for greater flexibility
- evidence that conditions that are at higher risk in un-neutered animals do not occur at a greater prevalence. Also would need to be a one shot procedure or I can see breakthroughs
- doesn't require boosters
- infrequent dosing e.g. once only or once every 5-10 years
- Should have a response based on welfare of the individual animal or if working across ecosystems reliability cost, delivery vs welfare impacts on individuals with any potential side effects.

### Fig 8b) Features which would increase their likelihood of using feline immunocontraceptives in the future should they become available and licensed

- evidence for management of feral cat populations
- How long does it last
- Proven efficacy, safety and reduction in cancers
- Lifetime cost cheaper than desexing
- Lower costs associated in using this particular method compared to routine surgical desexing.
- repeat injections not required
- price
- published proof of efficacy
