# Supplementary material for: Veterinarian attitudes towards immunocontraceptive use in cats
Source: Aust Vet J. 2026 Aug 6;104(10):701–9. doi: 10.1111/avj.70113 (PMC13631546; doi:10.1111/avj.70113)
Supplement: Supplementary file 2 [file AVJ-104-701-s002.docx]

Assessing veterinarian attitudes towards immunocontraceptive use in cats

Start of Block: Default Question Block

1 **Assessing veterinarian attitudes towards immunocontraceptive use in cats** Thank you for participating in this survey to assess veterinary attitudes towards immunocontraceptive use in cats. Please make sure that you have read the plain language statement before participating. We appreciate your feedback. This study will take approximately 10 minutes to complete

2 Immunocontraception is a technique that uses the host immune system to induce a contraceptive effect. This involves the use of antigens and consequently antibodies to target different aspects of gamete production and reproductive function to induce infertility. There have been several immunocontraceptives developed for use in animals, including GonaCon™, SpayVac™, Bopriva™ and Equity™. Immunocontraception has been proposed as a future contraceptive option for use in cats as an alternative to surgical sterilisation. Such use may be beneficial for population management of stray cat populations in regions with overabundant cat populations and administration of the immunocontraceptive may be delivered by a vaccine. Immunocontraceptives may also be an attractive contraceptive option for owners who may be unwilling to surgically desex their cats. There are several constructs for an immunocontraceptive. The immunocontraceptive vaccine may be in the form of purified reproductive antigen e.g. gonadotropin releasing hormone (GnRH) or Zona Pellucida (ZP) fused to an adjuvant. Alternatively, the immunocontraceptive may be virally delivered (for example with feline herpesvirus) as part of a combination contraceptive-vaccine capable of protecting against feline pathogens and pregnancy concurrently.

| Page Break |  |
| --- | --- |

Q1 In which country do you primarily practice small animal veterinary medicine?

- Afghanistan (1)
- Albania (2)
- Algeria (3)
- Andorra (4)
- Angola (5)
- Antigua and Barbuda (6)
- Argentina (7)
- Armenia (8)
- Australia (9)
- Austria (10)
- Azerbaijan (11)
- Bahamas (12)
- Bahrain (13)
- Bangladesh (14)
- Barbados (15)
- Belarus (16)
- Belgium (17)
- Belize (18)
- Benin (19)
- Bhutan (20)
- Bolivia (21)
- Bosnia and Herzegovina (22)
- Botswana (23)
- Brazil (24)
- Brunei Darussalam (25)
- Bulgaria (26)
- Burkina Faso (27)
- Burundi (28)
- Cambodia (29)
- Cameroon (30)
- Canada (31)
- Cape Verde (32)
- Central African Republic (33)
- Chad (34)
- Chile (35)
- China (36)
- Colombia (37)
- Comoros (38)
- Congo, Republic of the... (39)
- Costa Rica (40)
- Côte d'Ivoire (41)
- Croatia (42)
- Cuba (43)
- Cyprus (44)
- Czech Republic (45)
- Democratic Republic of the Congo (47)
- Denmark (48)
- Djibouti (49)
- Dominica (50)
- Dominican Republic (51)
- Ecuador (52)
- Egypt (53)
- El Salvador (54)
- Equatorial Guinea (55)
- Eritrea (56)
- Estonia (57)
- Ethiopia (58)
- Fiji (59)
- Finland (60)
- France (61)
- Gabon (62)
- Gambia (63)
- Georgia (64)
- Germany (65)
- Ghana (66)
- Greece (67)
- Grenada (68)
- Guatemala (69)
- Guinea (70)
- Guinea-Bissau (71)
- Guyana (72)
- Haiti (73)
- Honduras (74)
- Hong Kong (S.A.R.) (75)
- Hungary (76)
- Iceland (77)
- India (78)
- Indonesia (79)
- Iran (80)
- Iraq (81)
- Ireland (82)
- Israel (83)
- Italy (84)
- Jamaica (85)
- Japan (86)
- Jordan (87)
- Kazakhstan (88)
- Kenya (89)
- Kiribati (90)
- Kuwait (91)
- Kyrgyzstan (92)
- Lao People's Democratic Republic (93)
- Latvia (94)
- Lebanon (95)
- Lesotho (96)
- Liberia (97)
- Libyan Arab Jamahiriya (98)
- Liechtenstein (99)
- Lithuania (100)
- Luxembourg (101)
- Madagascar (102)
- Malawi (103)
- Malaysia (104)
- Maldives (105)
- Mali (106)
- Malta (107)
- Marshall Islands (108)
- Mauritania (109)
- Mauritius (110)
- Mexico (111)
- Micronesia, Federated States of... (112)
- Monaco (113)
- Mongolia (114)
- Montenegro (115)
- Morocco (116)
- Mozambique (117)
- Myanmar (118)
- Namibia (119)
- Nauru (120)
- Nepal (121)
- Netherlands (122)
- New Zealand (123)
- Nicaragua (124)
- Niger (125)
- Nigeria (126)
- North Korea (127)
- Norway (128)
- Oman (129)
- Pakistan (130)
- Palau (131)
- Panama (132)
- Papua New Guinea (133)
- Paraguay (134)
- Peru (135)
- Philippines (136)
- Poland (137)
- Portugal (138)
- Qatar (139)
- Republic of Moldova (141)
- Romania (142)
- Russian Federation (143)
- Rwanda (144)
- Saint Kitts and Nevis (145)
- Saint Lucia (146)
- Saint Vincent and the Grenadines (147)
- Samoa (148)
- San Marino (149)
- Sao Tome and Principe (150)
- Saudi Arabia (151)
- Senegal (152)
- Serbia (153)
- Seychelles (154)
- Sierra Leone (155)
- Singapore (156)
- Slovakia (157)
- Slovenia (158)
- Solomon Islands (159)
- Somalia (160)
- South Africa (161)
- South Korea (162)
- Spain (163)
- Sri Lanka (164)
- Sudan (165)
- Suriname (166)
- Swaziland (167)
- Sweden (168)
- Switzerland (169)
- Syrian Arab Republic (170)
- Tajikistan (171)
- Thailand (172)
- The former Yugoslav Republic of Macedonia (173)
- Timor-Leste (174)
- Togo (175)
- Tonga (176)
- Trinidad and Tobago (177)
- Tunisia (178)
- Turkey (179)
- Turkmenistan (180)
- Tuvalu (181)
- Uganda (182)
- Ukraine (183)
- United Arab Emirates (184)
- United Kingdom of Great Britain and Northern Ireland (185)
- United Republic of Tanzania (186)
- United States of America (187)
- Uruguay (188)
- Uzbekistan (189)
- Vanuatu (190)
- Venezuela, Bolivarian Republic of... (191)
- Viet Nam (192)
- Yemen (193)
- Zambia (580)
- Zimbabwe (1357)

| Page Break |  |
| --- | --- |

Q2 In which year did you graduate from veterinary school?

- 1958 (1)
- 1959 (2)
- 1960 (3)
- 1961 (4)
- 1962 (5)
- 1963 (6)
- 1964 (7)
- 1965 (8)
- 1966 (9)
- 1967 (10)
- 1968 (11)
- 1969 (12)
- 1970 (13)
- 1971 (14)
- 1972 (15)
- 1973 (16)
- 1974 (17)
- 1975 (18)
- 1976 (19)
- 1977 (20)
- 1978 (21)
- 1979 (22)
- 1980 (23)
- 1981 (24)
- 1982 (25)
- 1983 (26)
- 1984 (27)
- 1985 (28)
- 1986 (29)
- 1987 (30)
- 1988 (31)
- 1989 (32)
- 1990 (33)
- 1991 (34)
- 1992 (35)
- 1993 (36)
- 1994 (37)
- 1995 (38)
- 1996 (39)
- 1997 (40)
- 1998 (41)
- 1999 (42)
- 2000 (43)
- 2001 (44)
- 2002 (45)
- 2003 (46)
- 2004 (47)
- 2005 (48)
- 2006 (49)
- 2007 (50)
- 2008 (51)
- 2009 (52)
- 2010 (53)
- 2011 (54)
- 2012 (55)
- 2013 (56)
- 2014 (57)
- 2015 (58)
- 2016 (59)
- 2017 (60)
- 2018 (61)
- 2019 (62)
- 2020 (63)
- 2021 (64)
- 2022 (65)

Q3 How familiar are you with immunocontraceptives? (With 1 being extremely unfamiliar and 10 being extremely familiar)

- 1 (33)
- 2 (42)
- 3 (43)
- 4 (44)
- 5 (45)
- 6 (46)
- 7 (47)
- 8 (48)
- 9 (49)
- 10 (50)

| Page Break |  |
| --- | --- |

Q4 What is your primary method of reproductive sterilisation in healthy male cats?

- Orchiectomy (removal of the testes) (1)
- Vasectomy (2)
- Intratesticular injection (3)
- Hormonal contraceptives (4)
- Contraceptive implant (ie: suprelorin) (5)
- Contraceptive vaccine (6)
- I do not perform reproductive sterilisation in healthy male cats (7)
- Other (8) __________________________________________________

Q5 Thinking about the reasons behind why you chose that primary reproductive method in question 4, please rank each statement based on how much you agree or disagree with it with 1 being strongly disagree and 7 being strongly agree.

|  | 1 (1) | 2 (2) | 3 (7) | 4 (8) | 5 (9) | 6 (10) | 7 (11) |
| --- | --- | --- | --- | --- | --- | --- | --- |
| This method was what I was taught at veterinary school (1) |  |  |  |  |  |  |  |
| This is the standard practice at my clinic (2) |  |  |  |  |  |  |  |
| This is what I have read in the current literature (3) |  |  |  |  |  |  |  |
| This is what I have learnt at a recent veterinary conference (4) |  |  |  |  |  |  |  |
| This is what is legally allowable in my country (5) |  |  |  |  |  |  |  |

| Page Break |  |
| --- | --- |

Q6 What is your primary method of reproductive sterilisation in healthy nonpregnant female cats?

- Ovariohysterectomy (removal of the ovaries and uterus) (1)
- Ovariectomy (removal of only the ovaries) (2)
- Hysterectomy (removal of only the uterus) (3)
- Uterine tube (salpinx, oviduct) ligation (4)
- Hormonal contraceptives (5)
- Contraceptive implant (ie: suprelorin) (6)
- Contraceptive vaccines (7)
- I do not perform reproductive sterilisation in healthy non-pregnant female cats (8)
- Other (9) __________________________________________________

Q7 Thinking about the reasons behind why you chose that primary reproductive method in question 6, please rank each statement based on how much you agree or disagree with it, with 1 being strongly disagree and 7 being strongly agree.

|  | 1 (1) | 2 (2) | 3 (3) | 4 (4) | 5 (5) | 6 (6) | 7 (7) |
| --- | --- | --- | --- | --- | --- | --- | --- |
| This method was what I was taught at veterinary school (1) |  |  |  |  |  |  |  |
| This is the standard practice at my clinic (2) |  |  |  |  |  |  |  |
| This is what I have read in the current literature (3) |  |  |  |  |  |  |  |
| This is what I have learnt at a recent veterinary conference (4) |  |  |  |  |  |  |  |
| This is what is legally allowable in my country (5) |  |  |  |  |  |  |  |

| Page Break |  |
| --- | --- |

Q8 In what context could you imagine a use for immunocontraceptives in cats? (choose as many options as you like)

- Routine desexing in small animal clinics (1)
- Sterilisation of cats in overabundant stray cat communities (2)
- Sterilisation of feral cats (3)
- I dont see a use for immunocontraceptives in cats (4)
- Other (5) __________________________________________________

Q9 If an immunocontraceptive was licensed for use in cats, what would be your main reason/s for NOT using immunocontraceptives as a sterilisation method in cats? (Please rank the statements below based on how much you agree or disagree with them, with 1 being strongly disagree and 7 being strongly agree).

|  | 1 (1) | 2 (2) | 3 (3) | 4 (4) | 5 (5) | 6 (6) | 7 (7) |
| --- | --- | --- | --- | --- | --- | --- | --- |
| Lack of familiarity with immunocontraception (1) |  |  |  |  |  |  |  |
| Concerns about product safety (2) |  |  |  |  |  |  |  |
| Concerns about product efficacy (3) |  |  |  |  |  |  |  |
| Concerns about longevity (4) |  |  |  |  |  |  |  |
| Product availability (5) |  |  |  |  |  |  |  |
| Cost (6) |  |  |  |  |  |  |  |
| Unlikely to use as I dont see a use for them (7) |  |  |  |  |  |  |  |
| Other (8) |  |  |  |  |  |  |  |

| Page Break |  |
| --- | --- |

Q10 In what context/s would you see value for a vaccine contraceptive as opposed to other reproductive management strategies? (choose as many options as you like)

- Reversibility of method (1)
- Ability to perform procedure without general anaesthetic (2)
- Ability to relieve post-operative pain (3)
- Ability to maintain natural behaviours (4)
- Use in stray cat populations to avoid need for surgical desexing resources (5)
- I do not see any value for the use of a vaccine contraceptive (6)
- Other (7) __________________________________________________

Q11 Concerning the routine desexing of MALE cats, how important is avoiding or suppressing sexual behaviours to you? (With 1 being extremely unimportant and 10 being extremely Important)

- 1 (0)
- 2 (1)
- 3 (2)
- 4 (3)
- 5 (4)
- 6 (5)
- 7 (6)
- 8 (7)
- 9 (8)
- 10 (9)

| Page Break |  |
| --- | --- |

Q12 Concerning the routine desexing of FEMALE cats, how important is avoiding or suppressing sexual behaviours to you? (With 1 being extremely unimportant and 10 being extremely Important)

- 1 (0)
- 2 (1)
- 3 (2)
- 4 (3)
- 5 (4)
- 6 (5)
- 7 (6)
- 8 (7)
- 9 (8)
- 10 (9)

| Page Break |  |
| --- | --- |

Q13 In your opinion, what is/are the main reason/s that owners give when electing not to desex their animals? (Please rank the reasons below based on how much you agree or disagree, with 1 being strongly disagree and 7 being strongly agree).

|  | 1 (1) | 2 (2) | 3 (3) | 4 (4) | 5 (5) | 6 (6) | 7 (7) |
| --- | --- | --- | --- | --- | --- | --- | --- |
| Cost of procedure (1) |  |  |  |  |  |  |  |
| Safety concerns (2) |  |  |  |  |  |  |  |
| Wanting to breed (3) |  |  |  |  |  |  |  |
| Age of animal (4) |  |  |  |  |  |  |  |
| Personal choice (5) |  |  |  |  |  |  |  |
| Unsure (6) |  |  |  |  |  |  |  |
| Other (7) |  |  |  |  |  |  |  |

Q14 In your opinion how likely is owner acceptability of vaccine immunocontraceptives? (With 1 being extremely unlikely and 10 being extremely likely)

- 1 (0)
- 2 (1)
- 3 (2)
- 4 (3)
- 5 (4)
- 6 (5)
- 7 (6)
- 8 (7)
- 9 (8)
- 10 (9)

Q15 In your opinion, how likely is breeder acceptability of vaccine immunocontraceptives? (With 1 being extremely unlikely and 10 being extremely likely)

- 1 (0)
- 2 (1)
- 3 (2)
- 4 (3)
- 5 (4)
- 6 (5)
- 7 (6)
- 8 (7)
- 9 (8)
- 10 (9)

Q16 What would be the major feature/s needed for you to choose immunocontraceptives over more traditional contraceptives i.e. surgical, implant etc. (Please rank the statements below based on how much you agree or disagree with them with 1 being strongly disagree and 7 being strongly agree).

|  | 1 (1) | 2 (2) | 3 (3) | 4 (4) | 5 (5) | 6 (6) | 7 (7) |
| --- | --- | --- | --- | --- | --- | --- | --- |
| More cost effective for client (1) |  |  |  |  |  |  |  |
| More cost effective for clinic (2) |  |  |  |  |  |  |  |
| Improved efficacy (3) |  |  |  |  |  |  |  |
| Reduced morbidities (4) |  |  |  |  |  |  |  |
| Easier to implement (5) |  |  |  |  |  |  |  |
| Ability to provide dual protection against both fertility and feline pathogens if immunocontraceptive is virally vectored (6) |  |  |  |  |  |  |  |
| I would never choose immunocontraceptives over traditional methods (7) |  |  |  |  |  |  |  |
| Other (8) |  |  |  |  |  |  |  |

| Page Break |  |
| --- | --- |

Display this question:

If If an immunocontraceptive was licensed for use in cats, what would be your main reason/s for NOT... = Concerns about product safety [ 7 ]

Or If an immunocontraceptive was licensed for use in cats, what would be your main reason/s for NOT... = Concerns about product safety [ 4 ]

Or If an immunocontraceptive was licensed for use in cats, what would be your main reason/s for NOT... = Concerns about product safety [ 5 ]

Or If an immunocontraceptive was licensed for use in cats, what would be your main reason/s for NOT... = Concerns about product safety [ 6 ]

Q17 What are the major safety concerns you have regarding the use of  immunocontraceptives? (Please rank the statements below based on how much you agree or disagree with them with 1 being strongly disagree and 7 being strongly agree).

|  | 1 (1) | 2 (2) | 3 (3) | 4 (4) | 5 (5) | 6 (6) | 7 (7) |
| --- | --- | --- | --- | --- | --- | --- | --- |
| Injection site reactions (1) |  |  |  |  |  |  |  |
| Behavioural changes (2) |  |  |  |  |  |  |  |
| Waning efficacy over time (3) |  |  |  |  |  |  |  |
| Vaccine components (4) |  |  |  |  |  |  |  |
| Other safety concerns (5) |  |  |  |  |  |  |  |

Q18 Based on your current understanding, how likely are you to choose an immunocontraceptive for reproductive sterilisation of cats if one was licensed? (With 1 being extremely unlikely and 10 being extremely likely)

- 1 (0)
- 2 (1)
- 3 (2)
- 4 (3)
- 5 (4)
- 6 (5)
- 7 (6)
- 8 (7)
- 9 (8)
- 10 (9)

Q19 What would increase the likelihood of you using immunocontraceptives in cats in the future? (Please rank the statements below based on how much you agree or disagree with it, with 1 being strongly disagree and 7 being strongly agree).

|  | 1 (1) | 2 (2) | 3 (3) | 4 (4) | 5 (5) | 6 (6) | 7 (7) |
| --- | --- | --- | --- | --- | --- | --- | --- |
| Increase in peer reviewed studies (1) |  |  |  |  |  |  |  |
| Promotion through trusted pharmaceutical brands (2) |  |  |  |  |  |  |  |
| Requests from clients (3) |  |  |  |  |  |  |  |
| Seminars and conferences on the topic of immunocontraceptives (4) |  |  |  |  |  |  |  |
| Anecdotal evidence from other practitioners (5) |  |  |  |  |  |  |  |
| Ease of use for large scale community cat population management compared to surgical desexing (6) |  |  |  |  |  |  |  |
| Other (7) |  |  |  |  |  |  |  |

End of Block: Default Question Block
